# The Mouth Mirror

**Published:** 1897-04

**Authors:** 


ARTICLE X.

### THE MOUTH-MIRROR.

To my mind the month-mirror is by far the most useful instrument to be found on the operating table of the dental surgeon. So varied and numerous are its uses that it may be referred to as a veritable *multum in parvo.* Not only is it useful to reflect to the eye of the operator the surface upon which he is working, but it also serves to correct and condense the light upon this surface. It may be employed as a tongue-depressor, a cheek-distender, a mouth dilator, as an assistant to hold pieces of gold or amalgam while being condensed to place, to hold pellets of absorbent material, and even as a matrix. Or it may often serve in several of these capacities at one time.

For example: When a patient is seated in the chair, my first move is to take the mouth mirror in my left hand, and there it remains, serving in all the capacities above mentioned from time to time. If by any chance it be mislaid, I cannot proceed until it is found and restored to my hand. To me, therefore, it is indeed the most useful of instruments. I do not know whether I find more uses for the mouth-mirror than other operators or not, but there is one method of using it to which every operator should accustom himself, and if I can, by speaking of it here, cause any one who has not done so before, to school himself to so use it, I shall have done something worth the doing.

I refer to the most common of all its uses, that of reflecting the surfaces of points upon which we are operating. In excavating and filling cavities in the superior bicuspids and molars, on the morsal, mesial, distal or palatal surfaces, or upon the palatal, mesial and distal surfaces of the superior anterior teeth, I never see anything but the reflected image of the tooth. And my habit of so operating has saved me many a tired, aching back, and perhaps by this time a permanent deformity in a crooked spine and contracted, diseased chest.

In operating upon the class of cavities just mentioned, the chair should be low and tilted back but slightly, the operator should stand upright by the side of the patient's head, and to the rear of the chair, so as to be entirely free from contact with the body of the patient. Holding the mirror in the left hand, the arm is rested lightly upon the arm-support attached to the side of the head-rest, in which posture the glass may be held steadily in any desired position or angle, both reflecting the image of the tooth to the eye and directing the rays of light into the cavity. This position gives the operator the greatest freedom of movement of the right arm and shoulder, which is not the case where the body is bent over to the right to look directly into a cavity in the upper teeth, for then the shoulder and upper arm are so cramped as to be entirely ineffective, and only the wrist is free to move. Then, again, in this latter position, the eyes are hampered in their vision, and especially in their power to accurately gauge distances, by being brought into a vertical or horizontal position.

When looking into the mouth-mirror, at the image of a tooth which is between the eye and the mirror, the apparent distance of the tooth is increased by the distance between the mirror and the tooth, which enables the operator to obtain a focal distance more agreeable to the eye than the object itself would give. Consequently, the eyes endure the strain without exhaustion. A position above and back of the patient also gives the operator escape in a large measure from the poison of foul breaths. Aside from these considerations of greater efficiency and comfort of the operator, it is disgusting to see a man lay himself all over a patient in order to see directly into a cavity in a superior tooth.

Now this instrument being one that is used constantly in the mouth, and in so many mouths, being placed in direct contact with the mucous membrane, often in a diseased condition, and bathed in saliva always more or less vitiated, it follows that the mouth-mirror should receive the most careful attention as to its cleanliness and asepsis. Unfortunately, mirrors are so constructed as to afford lodgment for septic material in abundance, and are difficult, because of their construction, to render thoroughly aseptic. The ideal mirror in this respect is yet to appear, and he who brings forth a mouth-mirror fulfilling every requirement of asepsis will confer a great boon upon the dentist and a greater one upon the patient.

An ideal mirror would be a plain disc of some hard metal capable of receiving a high polish that could be renewed by the dentist himself by repolishing as needed. Such a mirror could be boiled in water and rendered aseptic in five minutes. But there is no metal that will receive such a polish as is necessary to reflect an unbroken image and give the depth and perfection of a glass mirror.

The nearest approach to a solution of the problem that I have seen is the mirror made with a screw-ring to hold the glass in the frame. This ring may be taken off, releasing the glass, which, when damaged, may be replaced by a new one, The frame may then be boiled to render it aseptic before another glass is replaced. To avoid the oxidation of the ring thread by saliva and antiseptic solutions used to disinfect the glass and frame while together, thus making it impossible to remove it, and also to seal up all crevices into which septic matter could penetrate, I adopted the plan of melting paraffin wax into the empty frame and over the threads of the screw, and while still fused setting in the glass and screwing down the ring, thus sealing it up effectually against the entrance of anything fluid or solid. The same plan may be followed profitably with the ordinary mirror by turning up the edge of the frame, removing the glass and resetting it in a bed of paraffin or cement, and reburnishing the upturned edge of the frame back against the glass.

To keep any kind of a mouth-mirror reasonably aseptic I

have a small vessel like a watch-crystal or other shallow dish on my operating case constantly filled with electrozone, a solution of chlorides, and a powerful antiseptic. After each patient, and frequently during the sitting of one patient, I dip the glass and such parts of the frame and handle as come in contact with the mouth into this solution and wipe oft thoroughly. Before doing so, however, I of course wash off any visible contamination with water. I use the full strength of electrozone, called dental meditrina. As a further precaution I keep in the drawer of my cabinet, where my mirrors are, a slab of plaster of Paris and sand saturated with Formaldehyd, as recommended by Dr. Cassidy a few years ago.

But in spite of all the precautions it is possible to adopt with a glass mirror, such as we are compelled to use for want of anything better, we may still be quite sure that it is *not* thoroughly asceptic at any time. Still it may be rendered at least innocuous by taking the precautions I have mentioned, and it is our duty to be religiously attentive to every detail of cleanliness that will tend to protect our patients who trust themselves to our care from anything septic or unclean.—*Dental Digest.*

---

